# Evaluating the feasibility of Preliminary Randomised Evaluation of Singing in Dementia (PRESIDE) in community group settings

**DOI:** 10.1186/s40814-026-01886-8

**Published:** 2026-07-22

**Authors:** Linda Elizabeth O’Raw, Becky Dowson, Martin Orrell, Orii McDermott, Lee Haywood, Phillip Bath, Lisa Woodhouse, Justine Schneider

**Affiliations:** 1 Institute of Mental Health, University of Nottingham Innovation Park, Jubilee Campus, Triumph Road, Nottingham, NG7 2TU UK; 2 Cambridge Institute for Music Therapy Research, Angela Ruskin University, Cambridge, UK; 3 Stroke Trials Unit, School of Medicine, University of Nottingham, Queen’s Medical Centre, Derby Road, Nottingham, NG7 2UH UK; 4 Law and Social Sciences Building, University of Nottingham, University Park, Nottingham, NG7 2RD UK

**Keywords:** Community singing, Dementia, Feasibility study, Intervention, Quality of life, Participant engagement

## Abstract

**Background:**

Community singing interventions have been explored as potential therapeutic activities for people with dementia and their carers. This feasibility study aimed to evaluate the practicality, engagement, and preliminary outcomes of such an intervention.

**Methods:**

A total of 72 participants (36 dyads) were recruited across three sites. Participants were randomly assigned to an intervention group, which engaged in community singing sessions, or a waiting list control group. Demographic characteristics were balanced between groups, and baseline musical engagement was low. Recruitment challenges and varying site fidelity were noted.

**Results:**

The intervention demonstrated mixed feasibility, with patient-reported outcome measures being practical and successfully administered. Descriptive trends favouring the intervention group were observed on some measures relating to quality of life, well-being, and daily activities, although the study was not powered to evaluate effectiveness. Retention at T1 was 78% in both arms; at T2, retention was 66.7% in the intervention arm and 77.8% in the control arm.

Notably, post-intervention, all sites transitioned to independent community singing groups or secured funding for continued activities, suggesting acceptability and ongoing participant interest.

**Conclusions:**

The study highlights the possible areas warranting further investigation of community singing interventions for people with dementia and their carers, with descriptive trends observed in selected quality-of-life and well-being measures. However, challenges such as recruitment and control condition engagement must be addressed in future research. Larger sample sizes and extended follow-up periods are necessary to evaluate intervention effectiveness and longer-term implementation outcomes.

**Trial registration:**

Funded by NIHR Research for Patient Benefit – Social Care (NIHR202982) and has been registered with ISRCTN (10201482) and IRAS (Project ID: 313298). The University of Nottingham is the trial sponsor.

## Key messages regarding feasibility

- What uncertainties existed regarding the feasibility?
  Key uncertainties related to the feasibility of recruitment, acceptability of the waiting list control design, consistency of intervention delivery across sites, participant retention, and the long-term sustainability of community singing groups for people living with dementia and their carers.
- What are the key feasibility findings?
  The study demonstrated that community singing sessions could be delivered across multiple community sites with acceptable retention, intervention adherence, and completion of patient reported outcome measures. However, recruitment represented a substantial feasibility challenge, with only 45% of the target sample achieved despite extensive recruitment efforts. Descriptive outcome trends and continued participant engagement following the intervention suggest that further investigation within a fully powered trial may be warranted.
- What are the implications of the feasibility findings for the design of the main study?
  The findings indicate that a future definitive trial would require substantial refinement of recruitment procedures, consideration of alternative comparator designs, clearer standardisation of core intervention components, and strategies to support consistent intervention fidelity across sites. Future studies would also benefit from larger sample sizes, extended follow-up periods, and embedded sustainability planning within community delivery settings.

## Background

Community-based interventions are vital for improving the social, emotional, and practical aspects of life for people with dementia and their carers, helping them navigate the challenges of the condition more effectively [1, 11, 21, 22]. Community-based music interventions provide opportunities for social interaction, reducing feelings of loneliness and fostering a sense of belonging [2, 9], including singing groups online [7]. Further, engaging in meaningful activities is thought to support cognitive function and emotional health [1] while addressing agitation [21]. Moreover, community-based music interventions help integrate people with dementia into societal activities, challenging stigma and promoting a more inclusive and dementia-friendly environment [1, 18]. Interventions involving people with dementia and their carers in community singing activities may empower them to remain active and engaged, fostering a sense of purpose and autonomy [1, 14, 18].

Existing evidence comes from small studies and is inconsistent, with some reporting no changes in standardised measures of quality of life, mood, or neuropsychiatric symptoms of dementia, despite positive qualitative findings [1, 4, 5]. Reliable evidence of benefit could inform social care strategy, attract additional funding for group singing initiatives, broaden options for social prescribers, and potentially alleviate pressure on health and social care services by enhancing the resilience of those with dementia and their caregivers in the community. Consequently, there is a need for rigorous, randomised controlled trial evidence to measure the effects of group singing on individuals with dementia and their caregivers.

This feasibility study seeks to evaluate the practicality of implementing community singing groups and explore possible trends relating to quality of life and the emotional, cognitive, and social well-being of both people with dementia and their carers.

## Aim

The purpose of the Preliminary Randomised Evaluation of Singing in Dementia (PRESIDE) study was to test the feasibility of conducting a pragmatic, randomised trial of community singing in dementia with a waiting list control group. The key feasibility indicators were recruitment, retention after 3 months, and the acceptability of the waiting list control trial design to participants. The secondary objective of the study was to examine the impact of group singing on people with dementia and their carers, using a range of standardised patient reported outcome measures, including quality of life and well-being.

## Methods and design

The study was a two-arm, randomised controlled, feasibility trial of community group singing using a waiting list control. Each dyad, consisting of a main participant (person with dementia) and a secondary participant (carer), was randomised into a control arm or intervention arm. The control arm waited up to 12 weeks before beginning singing sessions, whereas the intervention arm joined the singing session immediately post-baseline measures, for a minimum 10-week period. The study was carried out in three sites in the East Midlands of England. For anonymity, they are referred to as sites A, B, and C.

In keeping with the study design, a range of patient reported outcome measures (PROMs) were used that reflected the well-being of the person with dementia and the care partner, as well as the relationship of this dyad. This battery was informed by the results of a study of online singing during the COVID pandemic [7]. We also included measures of cognition to monitor disease progression, although we did not hypothesise that singing would be a disease-modifying intervention; rather, we predicted that its main effect would be on quality-of-life measures.

PROMs were measured at baseline (T0), 3 months post-baseline (T1), and 6 months post-baseline (T2) and completed within the home of the person with dementia. Control arm participants were offered singing intervention sessions at T1. Hence, all participants, regardless of randomised arm, had access to singing between months 3 and 6 of the study. Demographic data (age, sex, ethnicity, education, musical/singing experience) were collected at T0.

PROMs used included instruments to assess quality of life (DEMQOL/DEMQOL Proxy; [16]), social independence (Engagement and Independence in Dementia Questionnaire; [17]), dementia-specific outcomes (Dementia Core Outcome Set; [15]), loneliness (Three-item Loneliness Scale; [12]), musical engagement (Musical Engagement Questionnaire 15-item version; [20]), well-being (Short Warwick-Edinburgh Mental Wellbeing Scale; [19]), depression (Geriatric Depression Scale; [23]), and cognition (Mini Mental State Examination; [8]).

At baseline, PROMs were collated per dyad. They took approximately 2 h to complete. Post baseline, two researchers and delivery staff who were ‘blind’ to condition were assigned to administer the PROMs. This worked well and reduced the time taken to complete all PROMs.

### Participants

Participants who lacked capacity to provide informed consent at recruitment were excluded to ensure ethical integrity. Consent was treated as an ongoing process, with continuous monitoring of participants’ capacity. When a participant’s capacity deteriorated, a consultee was appointed to provide guidance on their continued involvement. If the participant or consultee expressed a wish to withdraw, participation was promptly terminated, retaining the data collected to date, ensuring the study remained ethically sound and voluntary.

Other exclusion criteria included significant hearing impairments; simultaneous participation in other interventional studies; and history of severe mental illness, alcohol, or drug addiction.

At the start of the project, which impacted the first site only, participants with dementia who had been in other singing groups in the past 6 weeks were excluded from the study. However, due to slow recruitment and given that the study’s purpose was to test feasibility criteria rather than outcomes for participants, a substantial amendment was made in February 2023 to include participants who had engaged with singing within the past 6 weeks.

The inclusion criteria for people with dementia were as follows: a formal diagnosis of mild to moderate dementia; being at least 18 years old; and having a carer who spent more than 2 h per week with them. To be included, participants also had to be willing to join a singing group and attend weekly; be able to give informed consent; and be able to speak English. Similarly, carers were required to speak and understand English; to be willing to give informed consent; and to be willing, in principle, to attend the singing group regularly for at least 10 weeks.

### Intervention

Preliminary online singing sessions following a similar intervention protocol were conducted during the COVID pandemic, delivering 30 sessions. Through this experience, the research team gained insights into the effective balance of warm-up exercises, conversation, and singing and refined the intervention protocol for PRESIDE [6]. For the present (face to face) study, three experienced community singing group leaders were recruited to run the sessions. They were provided with a guidance manual that describes the essential and optional elements of the intervention, while leaving them free to use their skills and experience (cf. Gold et al., [10]). A song book was produced to support the sessions, but it was not compulsory.

Singing sessions ran for 120 min (approximately 30 min of gentle exercise and vocal warm-ups, 60 min singing familiar and new songs or talking about music, and intermittent breaks accumulating to another 30 min). Fidelity scales for the intervention (see supplementary material) were administered at the start and again at 8 and 16 weeks. Participants randomised to start singing sessions right away were asked to attend weekly for 10 weeks. After this time, they could continue with the group if they wished, and the participants in the control arm joined the sessions.

### Sample size

The target sample size was 160 participants or 80 dyads, forty in each arm. It was calculated that a full trial with quality of life as its main PROM would need to recruit 360 analysable pairs and yield 80% power of achieving statistical significance at 5% level with 2-sided test if effect size ∆ = 0.3 (small). The two-arm equal-allocation feasibility trial aims to justify full trial but achieve significance only if ∆ exceeds 0.65 (large).

### Randomisation and ‘blinding’

Participants were stratified based on age with minimisation by age (± 80), sex and experience of singing, and computerised randomisation was performed in each stratum, so that it is weighted to account for these critical factors. This process was competed by LH.

The trial statistician (LW) and research staff administering 3 months (T1) and 6 months (T2) PROMs remained unaware of study arm allocation until analysis was complete. Research staff managing the project (BD, LO) were aware of study arm allocation because they attended singing sessions and conducted qualitative interviews.

### Recruitment

The study was open to recruitment from October 2022 to June 2023. Each site had approximately 3 months to recruit prior to the first singing session starting. In total, 30 weeks of the singing was provided to maximise on the window of opportunity available to recruit participants who could complete their 10-week intervention within this period.

Potential participants were screened over the telephone and pre-assessment home visits were arranged. Extensive recruitment efforts included advertising with social prescribers, engagement with Local Authorities and District Councils, distribution in day centres, visits to local GP surgeries, dementia cafes, post offices, pharmacies, libraries, and local television appearances. The ‘Join Dementia Research’ database was accessed to identify suitable participants in each local region (www.joindementiaresearch.nihr.ac.uk).

### Statistical analysis

The aim of the statistical analysis was to assess the following: the heterogeneity of the sample, retention rates in each arm of the study, and the performance of the PROMs in terms of their acceptability and validity, judged by missing data, as well as their apparent sensitivity to change, judged by variability and change over time. The statistician (LW) therefore generated descriptive data, summarising demographic and PROM, with mean and standard deviation (SD) for normally distributed variables; median and interquartile ranges (IQR) for skewed variables; and frequency and percentages (%) for categorical variables. These data were analysed by study arm at baseline, T1 (3 months), and T2 (6 months). Confidence intervals were reported to convey precision of estimates as the feasibility was not powered to detect effects.

## Results

### Demographic characteristics

The intervention and control arms were similar in age and ethnicity, with balanced demographic representation across both study arms on most variables (Table 1). The majority of the participants were White British.

**Table 1:** Sample characteristics

Baseline randomisation characteristics		Baseline: person with dementia		Baseline: carer	
		INT	Control	INT	Control
*N*		*n* = 18	*n* = 18	*n* = 18	*n* = 18
Age, years	Mean (SD)	77.3 (7.4)	75.9 (8.7)	67.5 (11.6)	68.8 (9.5)
Sex	*n* (%)	5 (27.8)	8 (44.4)	26 (72.2)	12 (66.7)
Ethnicity	*n* (%)				
	White	18 (100)	18 (100)	17 (94.4)	18 (100)
Relationship	*n* (%)				
	Spouse/partner			15 (83.3)	13 (72.2)
	Child			2 (11.1)	4 (22.2)
	Friend/other			1 (5.6)	1 (5.6)

### Recruitment

**Fig. 1:**
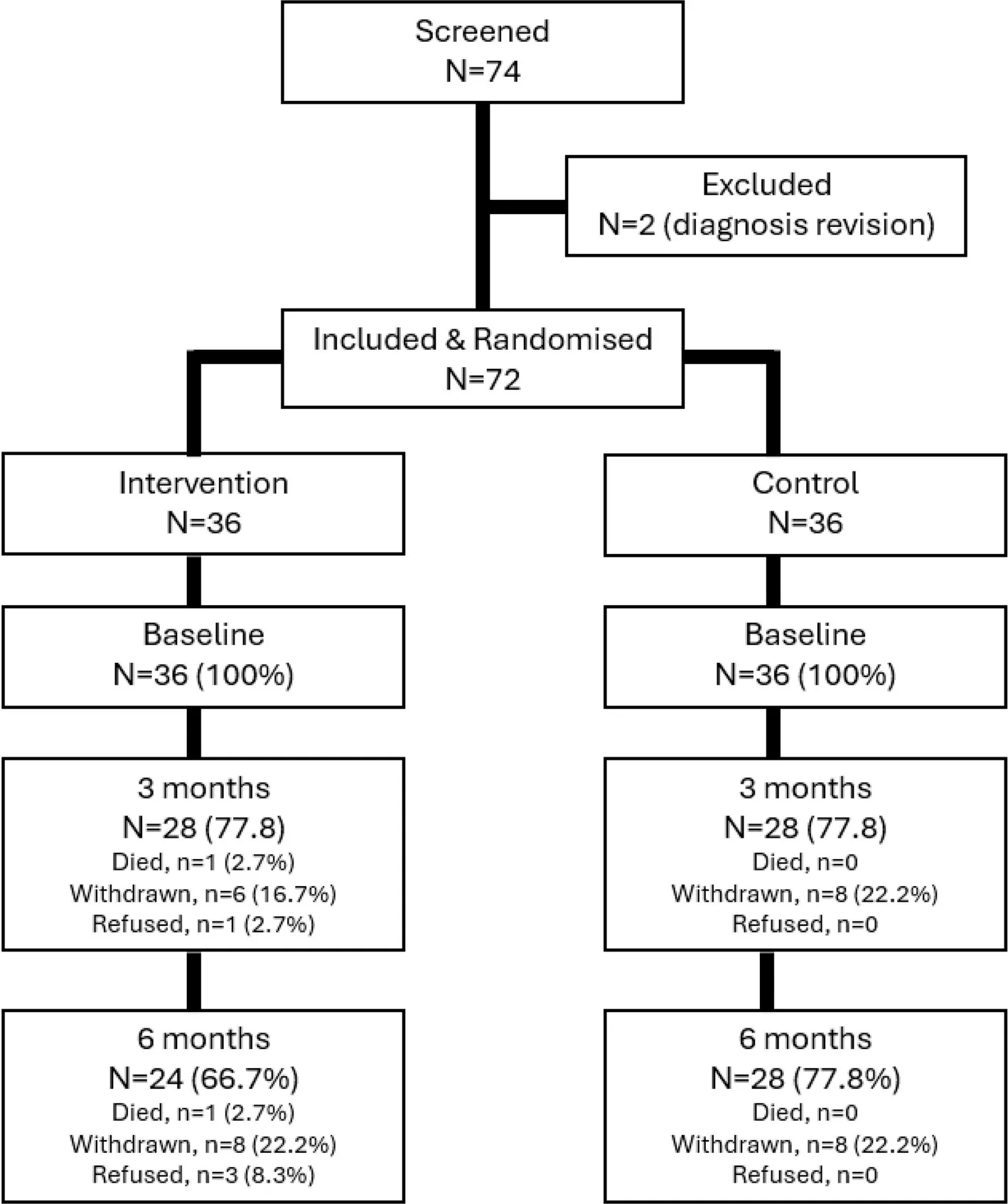
Participant flow diagram

The target of 160 participants (80 dyads) was not achieved. Despite strenuous recruitment efforts over more than 8 months, only 45% of the target sample was achieved: 72 participants (36 dyads) consented across three sites, A (*n* = 28; 14 dyads), B (*n* = 22; 11 dyads), and C (*n* = 22; 11 dyads) (see Fig. 1).

### Prior singing experience

Most participants (77.8%) indicated that they had ‘none’ or ‘not much’ prior singing experience (14 participants in both INT and Control arms). 22.3% indicated that they had ‘quite a lot’ or ‘extensive’ singing experience (4 participants in both INT and Control arms). Prior musical engagement was further measured via the Musical Engagement Questionnaire, for both the intervention and control arms. This showed MusEQ mean score (± SD) of 35.4 (6.7) in the intervention arm and 34.9 (7.2) in the control arm indicated a relatively low engagement with music prior to the study commencing.

### Retention

The retention rate at T1 (primary end point) was 78% in both arms. At T2, participant retention was lower in the intervention group (66.7%) than in the control group (77.8%). As shown in Fig. 1, the number of participants who withdrew were 22.2% in both the intervention and control arms.

### Attendance

Across all sites, 77.8% participants attended 5 or more sessions (50%) in the intervention group compared with 55% participants in the control arm. This includes 33% of participants in the control arm (waiting list) that attended no community singing sessions, compared to 6% of the intervention arm.

### Singing session fidelity

Table 3 shows Fidelity Checklist Adherence across three sites (A, B, and C). Higher percentages indicate fidelity to the intended model. Site A showed the highest adherence (86.3%) and Site B showed the lowest (65.61%). Overall, a high adherence to fidelity rate was achieved, observing a mean 78.8% across all sites (see Table 2).

**Table 2:** Fidelity checklist adherence by site

		% Fidelity checklist adherence			
	Facilitator	Site A	Site B	Site C	Item mean %
Essential Session Components	Welcome and check in	100	100	100	100
	Physical warm up	100	100	83.3	94.4
	Vocal warm Up	100	100	87.5	62.5
	Opening song	0	12.5	62.5	58.3
	Singing familiar songs	100	100	100	100
	Song choices offered	95.2	9.4	100	68.2
	Closing song	95.2	3	41.6	46.6
	Refreshments	100	100	100	100
	Totals	86.3	65.61	84.36	78.75

### Patient Report Outcome Measures (PROMs)

Baseline PROMs were completed by 100% of participants prior to randomisation. Follow-up PROMs were completed by 78% of all participants. This shows that the administration of PROMs was feasible.

The intervention and control arms did not differ significantly on any PROM. Table 3 shows the mean scores and standard deviations, 95% confidence intervals for the outcome measures at T1 (3 months) for people with dementia and carers, respectively.

**Table 3:** Three-month outcome measures for people with dementia and carers

	3-month outcome measures for people with dementia (T0–T1)			3-month outcome measures for carers (T0–T1)		
Variable	INT (*n* = 14)	Control (*n* = 14)	aMD (95% CI)	INT (*n*−13)	Control (*n* = 14)	aMD (95% CI)
	Mean (± SEM)			Mean (± SEM)		
DEMQOL	100.2 (10.1)	94.9 (13.1)	0.38 (−6.91, 7.67)			
DEMQOL-PROXY	97.1 (14.9)	91.1 (10.7)	2.29 (−6.42, 11.00)			
EID-Q: …Activities of Daily Living	18.3 (1.8)	17.3 (4.1)	1.11 (−1.12, 3.35)			
…Decision Making	13.1 (2.0)	11.7 (2.5)	1.25 (−0.29, 2.79)			
…Activity Engagement	10.5 (1.4)	8.9 (2.2)	1.22 (−0.30, 2.74)			
…Support	24.8 (3.1)	24.5 (2.9)	−0.12 (−2.58, 2.34)			
…Reciprocity	19.2 (3.7)	18.0 (4.6)	1.03 (−2.02, 4.08)			
Dementia core outcome set	37.1 (2.4)	36.0 (3.3)	0.81 (−1.25, 2.87)			
MMSE-brief	8.7 (3.1)	10.1 (3.5)	−0.64 (−2.47, 1.18)			
Three-item loneliness scale	3.8 (1.4)	4.1 (1.5)	−0.12 (−1.04, 0.80)	4.8 (1.8)	4.5 (1.7)	0.67 (−0.23, 1.56)
EQ-5D-5L HSUV	0.86 (0.26)	0.82 (0.16)	0.03 (−0.13, 0.19)	0.81 (0.17)	0.73 (0.23)	0.05 (−0.08, 0.18)
EQ-VAS	66.5 (15.9)	66.8 (12.2)	−1.07 (−11.4, 9.29)	75.0 (19.8)	75.9 (17.7)	5.07 (−5.86, 16.00)
Geriatric depression scale, GDS	1.3 (1.9)	3.3 (3.3)	−0.42 (−2.47, 1.63)	4.3 (2.9)	3.1 (2.3)	1.04 (−0.70, 2.78)
SWE Mental Wellbeing scale	56.6 (7.9)	53.2 (8.0)	2.84 (−2.54, 8.22)	48.7 (8.9)	52.4 (7.5)	−1.87 (−6.97, 3.24)
SIDECAR …(D) Direct impact				9.5 (5.2)	8.0 (3.8)	0.26 (−2.04, 2.56)
…(I) Indirect impact				6.2 (2.1)	6.3 (2.8)	0.58 (−0.85, 2.01)
…(S) Support and information				5.8 (2.7)	6.5 (2.6)	−0.36 (−2.04, 1.32)

Treatment effects were assessed at T1 using adjusted Mean Differences (aMD) with 95% Confidence Intervals (CI) (Table 3). The largest observed between-group difference was observed for DEMQOL (aMD = 0.38). For the intervention group, scores increased from 95.7 to 100.2 between baseline and T1 (aMD = −4.43, 95% CI −13.1 to 4.19). A similar slight increase was observed in the waiting list control group, with scores rising from 94.9 to 98.4 (aMD = −3.50, 95% CI −12.3 to 5.3). This pattern was also reflected in the DEMQOL-Proxy scores (see Table 3).

For people with dementia, Table 4 suggests that group community singing sessions showed descriptive trends favouring the intervention group on decision-making abilities (aMD = −2.90, 95% CI −5.31, −0.49), reciprocity in relationships (aMD = −3.41, 95% CI −6.72, −0.09), and mental well-being (aMD = −6.76, 95% CI −13.2, −0.29). Comparatively, no significant differences were observed in the waiting list control group for decision-making abilities, reciprocity, or mental well-being (95% CI not overlapping).

**Table 4:** Post singing session comparisons between intervention and control groups for people with dementia

Variable	INT Baseline v 3 months (T0vT1)			Control 3 months v 6 months (T1vT2)		
	Baseline (*n* = 18)	3 months (*n* = 14)	aMD (95% CI)	3 months (*n* = 14)	6 months (*n* = 14)	aMD (95% CI)
DEMQOL	95.7 (14.0)	100.2 (10.1)	−4.43 (−13.1, 4.19)	94.9 (13.1)	98.4 (11.3)	−3.50 (−12.2, 5. 23)
DEMQOL-PROXY	91.8 (17.5)	97.1 (14.9)	−5.29 (−17.9, 7.30)	91.1 (10.7)	96.8 (14.9)	5.75 (−16., 4.73)
EID-Q:						
Activities of Daily Living	16.7 (5.1)	18.3 (1.8)	−1.6 (−4.44, 1.24)	17.3 (4.1)	17.3 (3.3)	0.05 (−2.68, 2.78)
Decision Making	10.2 (4.2)	13.1 (2.0)	−2.90 (−5.31, 0.49)	11.7 (2.5)	11.2 (3.5)	0.50 (−1.67, 2.67)
Activity Engagement	9.3 (2.7)	10.5 (1.4)	−1.17 (−2.73, 0.40)	8.9 (2.2)	9.1 (2.0)	−0.21 (−1.74, 1.31)
Support	22.9 (4.5)	24,8 (3.1)	−1.89 (−4.67, 0.89)	24.5 (2.9)	24.7 (3.2)	−0.18 (−2.40, 2.05)
Reciprocity	15.8 (5.4)	19.2 (3.7)	−3.41 (−6.72, −0.09)	18.0 (4.6)	19.1 (4.0)	−1.07 (−4.14, 1.99)
MMSE-brief	8.6 (3.5)	8.7 (3.1)	−0.08 (−2.37, 2.21)	10.1 (3.5)	10.2 (3.7)	−0.07, (−2.66, 2.52)
Three-item loneliness scale	4.1 (1.4)	3.8 (1.4)	0.35 (−0.60, 1.30)	4.1 (1.5)	3.9 (1.4)	0.21 (−0.80, 1.23)
Dementia core outcome set	34.7 (4.8)	37.1 (2.4)	−2.37 (−5.13, 0.39)	36.0 (3.3)	36.9 (1.8)	−0.92 (−2.89, 1.05)
EQ-5D-5L HSUV	0.85 (0.19)	0.86 (0.26)	−0.01 (−0.16, 0.14)	0.82 (0.16)	0.83 (1.8)	−0.01 (−0.12, 0.10)
EQ-VAS	75.8 (18.2)	66.5 (15.9)	9,29 (−2,61, 21.20)	66.8 (12.2)	66.1 (19.7)	0.64 (−11.1, 12.34)
Geriatric depression scale, GDS	2.5 (2.6)	1.3 (1.9)	1.14 (−0.54, 2.81)	3.3 (3.3)	2.9 (3.4)	0.42 (−2.17, 3.01)
SWE mental well-being scale	49.8 (9.8)	56.6 (7.9)	−6.76 (−13.2, −0.29)	53.2 (8.0)	53.7 (8.5)	−0.48 (−6.47, 5.51)

## Discussion

The data gathered indicate that it was feasible to collect these PROMs from both people with dementia and their carers. Both people with dementia and carers were able to complete the assessments and measures without major issues. However, as a feasibility trial, the primary purpose of PRESIDE was not to determine intervention effectiveness, but rather to assess the practicality of recruitment, retention, intervention delivery, fidelity, outcome measurement, and acceptability of the trial design. Consequently, all outcome findings relating to PROMs should be interpreted cautiously and considered exploratory rather than confirmatory.

Whilethere was a lack of significant differences observed within and between groups, and no strong trends emerged, there were slight improvements in quality of life, well-being, and daily activities for people with dementia in the intervention group. These findings suggest that the intervention may warrant further investigation in a fully powered trial. Furthermore, longer follow-up periods may better explore longer-term outcomes.

The demographic profiles of the intervention and control arms were well-matched, with age and ethnicity being balanced across both groups. Predominantly, participants were White British, reflecting a relatively homogeneous sample in terms of ethnic background. This similarity ensures that demographic factors are unlikely to have introduced bias into the study’s outcomes. Singing experience and the Musical Engagement Questionnaire indicated that participants generally had relatively low levels of engagement with singing prior to starting the study.

Formal progression criteria were not pre-specified within the feasibility protocol. However, feasibility domains including recruitment, retention, acceptance of a waiting list control design, intervention fidelity, and PROM completion were evaluated descriptively to inform the viability and refinement of a future definitive trial. This feasibility study provides valuable insights into the feasibility and delivery of community singing interventions for people with dementia and their carers, but several limitations must be acknowledged. Firstly, the recruitment process presented challenges despite removing the exclusion criteria that prevented participants from joining with previous group singing experience. We found that using the Join Dementia Research database proved to be the most effective method of recruitment [13]. The slow rate of recruitment aligns with findings from other studies in this area [1, 21]. Other barriers to recruitment may include psychosocial, geographical, logistical, stigma-related, and financial impediments. For example, recruitment took place following lockdown restrictions from the COVID-19 pandemic, which could have discouraged participants [3]. Closer analysis of the pandemic’s impact on this study is undertaken in Dowson et al. [Dowson, B., O’Raw, L., Schneider, J., & McDermott, O.: "We always come out singing and skipping": The experience of people living with dementia and their companions taking part in group singing, submitted].

Recruitment feasibility was limited. Recruitment represented the principal feasibility challenge within the study. Despite extensive multi-site recruitment efforts conducted over more than eight months, only 45% of the target sample was achieved. This suggests that recruitment procedures, eligibility criteria, and/or broader contextual barriers require substantial refinement before progression to a definitive trial. Under-recruitment limited sample size, which would limit the statistical power of the study. This could have resulted in a Type 2 error, failing to show any statistical significance. Therefore, future studies could benefit from extending the recruitment timeframe and increasing the number of singing sessions to enable the recruitment target to be reached.

Although the waiting list control design was operationally feasible, evidence of reduced attendance and disengagement among participants initially allocated to delayed intervention access suggests that waiting periods may negatively influence participant motivation and intervention uptake. Importantly, this pattern related primarily to intervention attendance rather than study retention, which remained acceptable across both groups. These findings have important implications for the acceptability of waiting list comparator designs within future definitive trials [2, 21].

Retention in both arms was, overall, good, although higher retention rates were observed in the control group. When investigating further, outside factors, such as the complexities of living with dementia could be attributed to drop out rather than the impact of the intervention itself. The slight changes in retention rates between intervention and control arms do not adequately reflect the sustainability of the community singing intervention.

The study achieved high fidelity rates overall, showing many strong components (such as ‘Welcome and Check-in’, ‘Singing Familiar Songs’, ‘Refreshments’). Sites differed somewhat in ‘Song Choices Offered’ and using an ‘Opening Song’ or a ‘Closing Song’. It remains unclear if such differences between sites are likely to influence the intended PROMs, but this is a question which future studies could address. Post intervention, self-sustainability was observed across all sites in various ways. Our study showed one site transitioned to an independent singing group for people with dementia and their carers, managed by a charitable organisation, retaining its membership base who took responsibility for the breaktime drinks and snacks. Another site secured external funding with a national charity to continue singing activities for individuals with dementia and their carers. Our final site did not continue as a group, but all participants joined other local singing groups with the exception of two dyads; one dyad has started their own singing group in a care home while the other dyad is seeking something a little more local to them. This enduring engagement suggests that the participants found significant value and enjoyment in the sessions, which contributed to their continued participation beyond the study’s formal end. In addition to the potential cost-effectiveness, the self-sustainability of these groups indicates that a high level of acceptability and perceived benefit among the participants, indicating that community singing interventions foster lasting community engagement and personal satisfaction.

Variability in fidelity across sites may reflect differences in facilitator style, local delivery context, and participant preferences. While overall adherence was acceptable, future trials may require greater standardisation of core intervention components alongside preservation of facilitator flexibility. They may further benefit from exploring alternative comparator designs, such as active social interventions or shorter waiting periods, to minimise disengagement associated with delayed participation.

While retention, intervention adherence, PROM completion, and post-study sustainability indicators were encouraging, recruitment fell substantially below target despite intensive efforts. Therefore, the findings support the feasibility of intervention delivery and data collection procedures but indicate that recruitment methodology requires further optimisation before a definitive trial could proceed successfully. The findings support the feasibility and acceptability of delivering community singing within community settings, while highlighting several methodological challenges requiring refinement before a definitive trial.

## Conclusion

PRESIDE demonstrated that community singing interventions for people living with dementia and carers can be delivered within community settings with acceptable retention, fidelity, and outcome measure completion. However, substantial recruitment challenges and issues relating to waiting list control acceptability indicate that methodological refinements are required before progression to a fully powered definitive trial.

## References

[1] Baker FA, Pac Soo V, Bloska J, Blauth L, Bukowska AA, Flynn L, et al. Home-based family caregiver-delivered music and reading interventions for people living with dementia (HOMESIDE trial): an international randomised controlled trial. EClinicalMedicine. 2023;65:102224. 10.1016/j.eclinm.2023.102224. PMC10725050 38106552

[2] Coulton S, Clift S, Skingley A, Rodriguez J. Effectiveness and cost-effectiveness of community singing on mental health-related quality of life of older people: randomised controlled trial. Br J Psychiatry. 2015;207:250–5. 10.1192/bjp.bp.113.129908. 26089304

[3] Docherty S, Haskell-Ramsay CF, McInnes L, Wetherell MA. The effects of COVID-19 lockdown on health and psychosocial functioning in older adults aged 70 and over. Gerontol Geriatr Med. 2021;7:23337214211039976. 10.1177/23337214211039974. PMC8580486 34778495

[4] Dorris J, Rodakowski J. Music interventions for adults living with dementia. Am J Geriatr Psychiatry. 2024;32(3):312–4. 10.1016/j.jagp.2023.10.011. 37949803

[5] Dowson B, McDermott O, Schneider J. What indicators have been used to evaluate the impact of music on the health and wellbeing of people with dementia? A review using meta-narrative methods. Maturitas. 2019;127:26–34. 10.1016/j.maturitas.2019.06.001. 31351517

[6] Dowson B, Schneider J, Guo B, Bath PM, McDermott O, Haywood LJ, et al. Pilot randomised evaluation of singing in dementia (PRESIDE): protocol for a two-arm, parallel-group randomised controlled feasibility study with waiting-list control. Pilot Feasibility Stud. 2021;7(1):15. 10.1186/s40814-020-00759-y. PMC7787932 33407912

[7] Dowson B, Schneider J, McDermott O, Orrell M. Online singing groups for people with dementia: adaptation and resilience in the face of the COVID-19 pandemic. Dementia (London). 2023;22(7):1348–71. 10.1177/14713012231179262. PMC10261964 37294296

[8] Folstein MF, Folstein SE, & McHugh PR. “Mini-mental state”: A practical method for grading the cognitive state of patients for the clinician. J Psychiatr Res. 1975;12(3):189–198. 10.1016/0022-3956(75)90026-6. 1202204

[9] Freedman A, Nicolle J. Social isolation and loneliness: the new geriatric giants: approach for primary care. Can Fam Physician. 2020;66(3):176–182. https://www.ncbi.nlm.nih.gov/pubmed/32165464. PMC8302356 32165464

[10] Gold C, Eickholt J, Assmus J, Stige B, Wake JD, Baker FA, et al. Music interventions for dementia and depression in elderly care (MIDDEL): protocol and statistical analysis plan for a multinational cluster-randomised trial. BMJ Open. 2019;9(3):e023436. 10.1136/bmjopen-2018-023436. PMC6475205 30928926

[11] Harding AJE, Morbey H, Ahmed F, Opdebeeck C, Elvish R, Leroi I, et al. A core outcome set for nonpharmacological community-based interventions for people living with dementia at home: a systematic review of outcome measurement instruments. Gerontologist. 2021;61(8):e435–48. 10.1093/geront/gnaa071. PMC8599310 32583858

[12] Hughes ME, Waite LJ, Hawkley LC & Cacioppo JT. A short scale for measuring loneliness in largesurveys: Results from two population-based studies. Research on Aging. 2004;26(6):655–72. 10.1177/0164027504268574. PMC2394670 18504506

[13] Join Dementia Research. Register your interest in dementia research. National Institute for Health and Care Research. (n.d.). https://www.joindementiaresearch.nihr.ac.uk/.

[14] Osman SE, Tischler V, Schneider J. “Singing for the Brain”: a qualitative study exploring the health and well-being benefits of singing for people with dementia and their carers. Dementia (London). 2016;15(6):1326–39. 10.1177/1471301214556291. PMC5089222 25425445

[15] Reilly ST, Harding AJE, Morbey H, Ahmed F, Williamson PR, Swarbrick C, Leroi I, Davies L, Reeves D, Holland F, Hann M, & Keady J. What is important to people with dementia living at home? A set of coreoutcome items for use in the evaluation of non-pharmacological community-based health and social care interventions. Age and Ageing. 2020;49(4):664–71. 10.1093/ageing/afaa015.

[16] Smith SC, Lamping DL, Banerjee S, Harwood RH, Foley B, Smith P, et al. Development of a new measure of health-related quality of life for people with dementia: DEMQOL. Psychol Med. 2007;37(5):737–46. 10.1017/S0033291706009469. 17176501

[17] Stoner CR, Orrell M, Spector A. Psychometric properties and factor analysis of the Engagement and Independence in Dementia Questionnaire (EID-Q). Dement Geriatr Cogn Disord. 2018;46(3–4):119–27. 10.1159/000488484. 30179862

[18] Tamplin J, Thompson Z. How health-focused choirs adapted to the virtual world during the COVID-19pandemic: An international survey. The Arts in Psychotherapy. 2023;82:Article 101997. 10.1016/j.aip.2023.101997. PMC9846900 36686980

[19] Tennant R, Hiller L, Fishwick R, Platt S, Joseph S, Weich S, et al. The Warwick-Edinburgh Mental Well-being Scale (WEMWBS): development and UK validation. Health Qual Life Outcomes. 2007;5:63. 10.1186/1477-7525-5-63. PMC2222612 18042300

[20] Vanstone AD, Wolf M, Poon T, & Cuddy LL. Measuring engagement with music: Development of an informant-report questionnaire. Aging & Mental Health. 2016;20(5):474–84. 10.1080/13607863.2015.1021750. 25811870

[21] Veal BM, Dobbs D, Lee S, Bugos JA, Pyfrom MP, Boddupalli S, et al. Feasibility and acceptability of a group music intervention in memory care communities. J Appl Gerontol. 2022;41(6):1528–38. 10.1177/07334648221079118. 35341380

[22] Whitlatch CJ, Orsulic-Jeras S. Meeting the informational, educational, and psychosocial support needs of persons living with dementia and their family caregivers. Gerontologist. 2018;58(suppl_1):S58–73. 10.1093/geront/gnx162. 29361068

[23] Yesavage JA, Brink TL, Rose TL, Lum O, Huang V, Adey M, & Leirer VO. Development and validation of a geriatric depression screening scale: A preliminary report. J Psychiatr Res. 1982;17(1):37–49. 10.1016/0022-3956(82)90033-4. 7183759

